# The relationship of living arrangements and depressive symptoms among older adults in sub-Saharan Africa

**DOI:** 10.1186/1471-2458-13-682

**Published:** 2013-07-25

**Authors:** Brittany McKinnon, Sam Harper, Spencer Moore

**Affiliations:** 1 Department of Epidemiology, Biostatistics and Occupational Health, McGill University, Purvis Hall, 1020 Pine Avenue West, Quebec H3A 1A2, Montreal, Canada; 2 School of Kinesiology and Health Studies, Queen’s University, Kingston, Canada

**Keywords:** Older adults, Mental health, Living arrangements, Sub-Saharan Africa

## Abstract

**Background:**

Older adults in sub-Saharan Africa are increasingly facing the twin challenges of reduced support from their adult children and taking on new roles caring for orphans and vulnerable children. How these changes affect the mental health of older adults is largely unknown.

**Methods:**

We use data from the 2002–2003 World Health Surveys for 15 countries in sub-Saharan Africa to examine whether older adults who may be lacking adequate support through living alone or in skipped-generation households are at an increased risk of depressive symptoms compared to those living with at least one working-age adult. Using meta-regression, we also examine whether heterogeneity across countries in the prevalence of depressive symptoms or in the association between living arrangements and depressive symptoms is associated with HIV/AIDS prevalence and national economic status.

**Results:**

The pooled prevalence of depressive symptoms among older adults was 9.2%. Older adults living alone had a 2.3% point higher predicted prevalence of depressive symptoms compared to individuals living with at least one working-age adult (95% confidence interval: 0.2%, 4.4%). None of the country characteristics examined explained heterogeneity across countries in the relationship between living arrangements and depressive symptoms. However, there was some evidence suggesting a positive association between depressive symptom prevalence and the severity of a country’s HIV/AIDS epidemic.

**Conclusion:**

As depressive symptoms are known to be predictive of poor quality of life and increased mortality, it is important to address how health and social policies can be put in place to mitigate the potentially detrimental effects of solitary living on the mental health of older persons in sub-Saharan Africa.

## Background

In sub-Saharan Africa, families have traditionally been the primary source of care and support for older people, with the majority of older adults residing with adult children and their families [1]. These intergenerational living arrangements stem from strong cultural traditions of intergenerational reciprocity, as well as a near nonexistence of old age pension programs and other forms of social assistance that enable older people to live more independently [1-3]. Although the majority of older Africans continue to rely on younger family members for material and social support, there is growing evidence that traditional caring and support mechanisms are under increasing strain throughout the region [4,5]. Some evidence points to a weakening of traditional values resulting from the effects of development, modernization and increased educational opportunities for younger generations, while other research suggests that increasing poverty and economic hardship has left younger adults increasingly unable to provide adequate support for elders [6]. Furthermore, there is growing evidence that increased mortality of working-age adults from the HIV/AIDS epidemic is weakening the support networks of older people and leading to an increasing proportion of older adults living alone and in ‘skipped-generation’ households—households with an older adult and a young child in the absence of a working-age adult [7-9]. A recent study found that more than 20% of adults over the age of 60 were living in skipped-generation households in Lesotho, Rwanda, Uganda, and Malawi [10].

Depression is one of the most common medical conditions among older people and is a major public health concern in both high- and low-income countries. Depression and depressive symptoms among older adults are predictive of functional impairment, poor quality of life, increased use of health services, and increased mortality [11-15]. Common risk factors for depression among older adults include: low socioeconomic status, lack of social support, poor health, and being female [16]. There has been limited research in African settings on the determinants of depression or depressive symptoms among older adults. One of the only community-based studies, the Ibadan Study of Ageing in Nigeria, found a 12-month prevalence of major depressive disorder of 7.1% (95% CI: 5.9-8.3%) among people aged 65 and older [17]. In this study, women and individuals residing in urban areas had higher odds of depression. Depressed older people also had impaired quality of life and greater difficulty functioning at home and in social roles.

Research across many different societies has found that older people who live alone or are socially isolated are more likely to suffer from depressive symptoms [11-15]. Much less is known about the relationship between skipped-generation living arrangements and depression or depressive symptoms among older adults. There is evidence from China that older adults living with grandchildren in skipped-generation households were less likely to suffer from depressive symptoms compared with those living in single-generation households [15]. However, in the Chinese setting it is common for older adults to receive remittances from absent adult children in order to support themselves and their grandchildren. In settings where mortality due to HIV/AIDS is a substantial cause of skipped generation households, older people may be less likely to receive material support to care for themselves and dependent grandchildren. In this context, it is not known whether living with grandchildren would confer the same mental health benefits for older adults.

Using data from 15 sub-Saharan African countries, we examined whether older Africans who may be lacking adequate support as a consequence of living alone or in skipped-generation households report more depressive symptoms compared to those living in households with at least one working-age adult. We also examine whether the associations between living arrangements and depressive symptoms differ across countries, and whether this heterogeneity can be explained by country-level factors, such the severity of the HIV/AIDS epidemic and country economic status.

## Methods

### Data

We used data from the World Health Surveys (WHS), access to which was granted through a grant from the Canadian Institutes for Health Research (#191612). The WHS is a large cross-sectional study conducted in 70 high-, middle-, and low-income countries in 2002–2003. The surveys were designed to provide cross-nationally comparable assessments of socio-demographics, adult and child morbidity and mortality, risk factors, health care expenditures, and coverage of health interventions. Comprehensive information about the WHS can be found on their website (http://www.who.int/healthinfo/survey/en/). The World Health Surveys were approved by the World Health Organization’s ethical review process and cleared by ethics review committees in each country. Informed consent was obtained from all survey respondents [18]. For this study, we restricted our analyses to individuals aged 50 years and over living in the 15 continental sub-Saharan Africa countries that participated in the WHS: Burkina Faso, Chad, Congo, Cote d’Ivoire, Ethiopia, Ghana, Kenya, Malawi, Mali, Namibia, Senegal, South Africa, Swaziland, Zambia, and Zimbabwe. For these countries, the WHS consisted of face-to-face interviews of individuals living in private households. The age of 50 years has been considered an appropriate cut-off for studying older people in sub-Saharan Africa, as life expectancy is relatively low in the region and individuals normally become grandparents by that age [19,20]. We had a total sample size of 12,647 with country sample sizes ranging from 425 in Congo to 1166 in Mali. Samples are nationally representative for all countries except Congo and Côte d’Ivoire, where the survey was carried out in limited geographical areas due to civil unrest at the time.

### Measures

#### Outcome

Our outcome is depressive symptoms reported in the past 12 months, which was defined as a dichotomous variable based on the responses to five questions taken from the World Mental Health Survey (WMH) version of the Composite International Diagnostic Interview (CIDI) [21]. This simplified version of the full CIDI was specifically designed to be applicable in cross-cultural settings and has been validated across several different populations and countries [22]. The specific symptoms asked about were: 1) Have you had a period lasting several days when you felt sad, empty or depressed? 2) Have you had a period lasting several days when you lost interest in most things you usually enjoy such as hobbies, personal relationships or work? 3) Have you had a period lasting several days when you have been feeling your energy decreased or that you are tired all the time? We considered an individual to have experienced depressive symptoms in the past 12 months if they met three criteria: 1) Responded yes to at least two of three symptoms; 2) The period of sadness/loss of interest/low energy was present for more than two weeks; and 3) During the period of sadness/loss of interest/low energy, symptoms were present most of the day, nearly every day. This measure of depressive symptoms includes hallmark depressive symptoms, considers important duration and frequency criteria, and has been previously used in a large multi-country study covering all world regions [18].

#### Living arrangements

Older adult living arrangements were grouped into three types: 1) *Single*-*generation household*: older person living in a household without any individuals under the age of 50 years; 2) *Skipped*-*generation household*: older person living in a household with a child age 17 years or younger in the absence of an adult aged 18–49 years; and 3) *Multigenerational household*: older person living in a household with at least one adult aged 18–49 years. This information was obtained from the household rosters portion of the WHS household survey. Individuals present in the household roster included everyone currently living at the household address. The roster included each household member’s age, level of education, and marital status. No distinction with respect to the specific biological relationships of the household members to the older person were made since an exclusive focus on the parent/child bond when considering the living arrangements of older people is inappropriate in developing country contexts where strong community and extended family relations exist [23].

#### Individual-level covariables

We examined several variables as potential confounders of the relationship between living arrangements and depressive symptoms. Socio-demographic variables included: age, gender, marital status (married/cohabitating vs. divorced/widowed/single), urban/rural residence, education level (no education vs. some education), and socioeconomic status (SES). As in other low-income settings, SES was estimated using an asset-based index [24]. To estimate the index we used the approach developed by Ferguson et al. [25], which uses household ownership of assets (e.g., a bicycle, a radio), access to services (e.g., electricity, drinking water), and known predictors of income (e.g., age, education) to estimate a latent indicator of permanent household income. This measure of permanent income has been validated against reported household income and expenditures using household survey data from Greece, Peru, Indonesia, Nepal, and Pakistan [24,25]. Finally, we created a dichotomous variable indicating whether an individual had ever been diagnosed with at least one of the following chronic physical health conditions: asthma, arthritis, angina, and diabetes.

#### Country-level variables

We examined three country-level variables: HIV/AIDS prevalence rate, gross national income per capita (GNIpc), and maternal mortality rate (MMR). We hypothesized that older people living alone and in skipped generation households in countries with a greater burden of HIV/AIDS may be especially vulnerable to depressive symptoms because of the greater likelihood they may have reduced levels of material support for themselves and dependent children. HIV/AIDS prevalence rates for each country’s population age 15–49 were obtained from public reports produced by UNAIDS for the year 2002 [26]. Across 17 high- and low- and middle-income countries, GDPpc was shown to be positively correlated with the lifetime risk of a mood disorder [4]. As such, we also examined GNIpc in 2002 (in 2005 purchasing power parity adjusted international dollars), obtained from the World Bank’s 2005 World Development Indicators (WDI) report for all countries except Zimbabwe (which we excluded from this part of the analysis) [27]. Finally, MMR is the number of deaths of women from pregnancy-related causes per 100,000 live births and was also obtained from WDI. We use MMR as a proxy for the general quality of a country's health system [28]. Although mental health care across sub-Saharan Africa is in general severely under-resourced and under-prioritized, countries with more established health systems may provide somewhat better services for mental health conditions [5,29].

### Statistical analysis

We first assessed the relationship between living arrangements and depressive symptoms using separate logistic regression models for each country, adjusted for age, gender, marital status, urban/rural residence, education level, and wealth index. To facilitate interpretation for all models and to assess differences on the absolute probability scale, we reported average marginal effects calculated from the logistic coefficients [30]. For example, the marginal effect for skipped-generation living arrangement is interpreted as the estimated effect on the predicted prevalence of depressive symptoms of living in a skipped-generation household compared to living in a multi-generational household, averaged over the values of the other covariates in the model. To obtain an overall effect estimate and because the country-specific estimates lack precision due to small sample sizes, we pooled the country-specific estimates across the 15 countries using random-effects meta-analysis [31]. This approach estimates an overall effect by computing a weighted average of the country-specific estimates. In using random-effects meta-analysis, we assume our sample of countries represents a potentially random sample of all countries in sub-Saharan Africa and does not assume there is a common, homogeneous effect of exposure [31]. We measured heterogeneity in the magnitude of the effect estimates using the *I*^2^ statistic, which quantifies the percent of variation in the effect that is due to heterogeneity between countries rather than sampling variability. We consider an *I*^2^ value below 50% to be low, 50-75% moderate, and ≥75% high [32].

We then used random-effects meta-regression to assess whether heterogeneity between countries in the predicted prevalence of depressive symptoms was related to country-level variables (HIV prevalence, log(GNIpc), MMR), where the meta-regression estimates were weighted by the inverse of the standard errors of the prevalence estimates, thus placing greater weight on estimates with greater precision [31]. Using the same method, we also examined whether heterogeneity in the estimated marginal effects of the living arrangement variables on the predicted prevalence of depressive symptoms were related to country-level variables. The meta-regression is conducted at the country-level, regressing the country-specific effect estimates on the country-level predictor variables. These meta-regression analyses were weighted by the inverse of the standard errors of the marginal effect estimates. Sampling weights were incorporated into all models (except for Congo and Cote d’Ivoire where individuals were equally weighted).

Missing responses for the three main depressive symptom variables ranged from 5.8-6.4% across countries and approximately 11% of responses were missing for the frequency of depressive symptoms. To account for missing data we performed multiple imputation using the *mi impute chained* procedure in Stata 12, which uses an iterative multivariable regression procedure to generate distributions for each variable with missing data that are conditional on all other variables in the imputation models [33]. All variables with missing data that were used in the analyses were imputed and a total of 10 imputed datasets were generated. Estimates from the imputed datasets were combined using Stata’s *mi estimate* commands, which account for variation in estimates and standard errors across imputed datasets [33,34].

## Results

**Figure 1:**
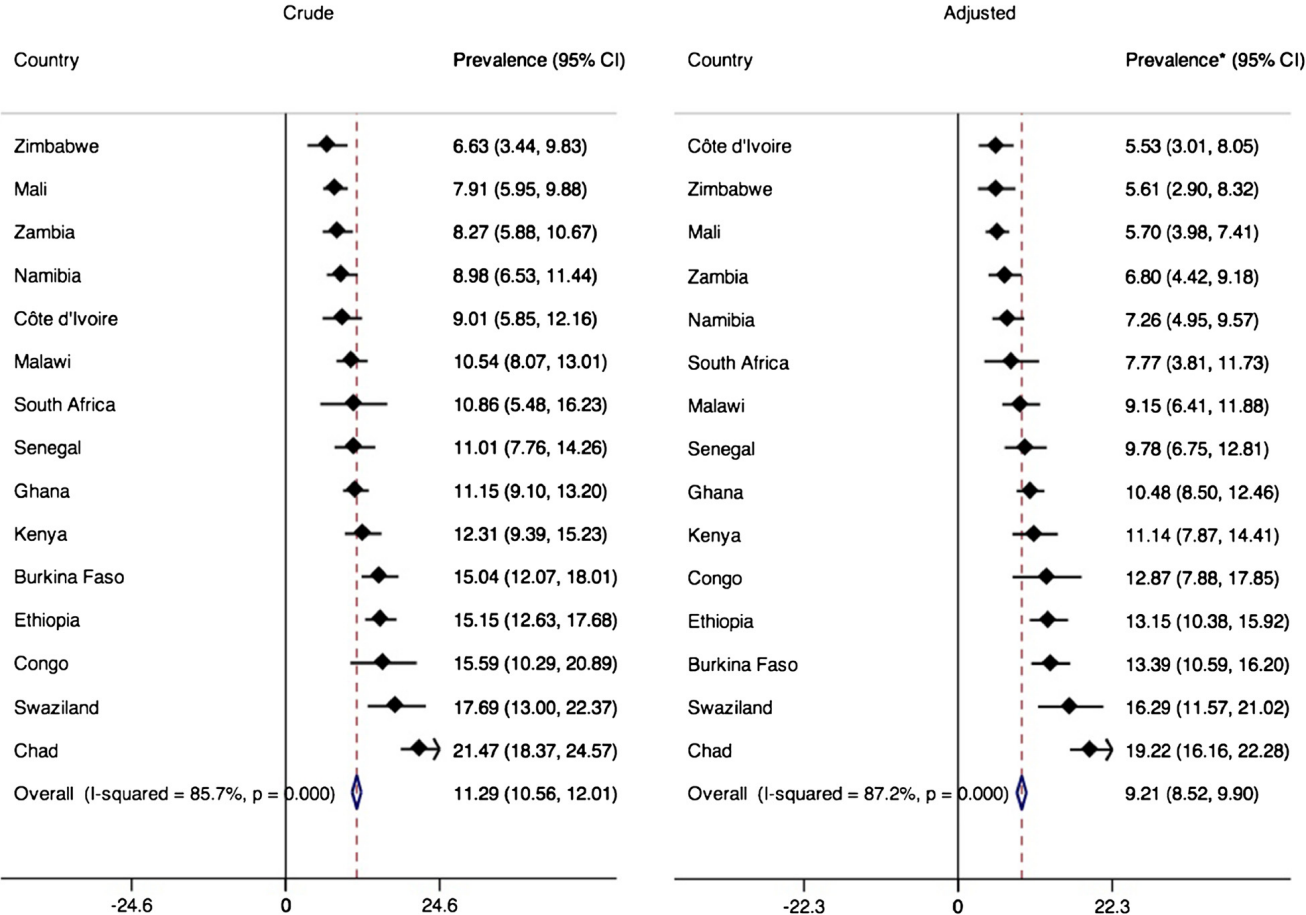
Random effects meta-analysis of the crude and adjusted predicted prevalence of depressive symptoms, WHS 2002-2003.

Table 1 presents descriptive characteristics for the study population. There was substantial heterogeneity across countries in the proportion of older adults residing in different living arrangements. Malawi had by far the highest prevalence of skipped generation households at 29%. This is in stark contrast to Mali and Senegal where the figure was less than 3%. In South Africa, Kenya and Malawi, 20% or more of older adults lived in single generation households, whereas in Mali and Senegal it was less than 2%. Only in a few countries (Kenya, South Africa, Swaziland, and Congo) was it more common for older adults to live in single-generation households than in skipped-generation households. Figure 1 shows the estimated crude and adjusted prevalence of depressive symptoms across countries. Based on the multivariate country-specific models, we found substantial between-country variation in the predicted prevalence of depressive symptoms among adults over the age of 50 years, ranging from 5.5% in Côte d’Ivoire to 19.2% in Chad. The overall covariate-adjusted random-effects estimate of the predicted prevalence of depressive symptoms was 9.2% (95% CI: 8.5, 9.9), slightly lower than the crude estimate of 11.3% (10.6, 12.0).

**Table 1:** Descriptive characteristics, WHS 2002-03

**Country**	**n**	**% Skipped generation household**	**% Single generation household**	**Mean age**	**% Male**	**% Urban**	**% Married**	**% No education**	**% Chronic disease**^**a**^	**GNIpc**^**b**^	**HIV/AIDS** **prevalence**^**c**^	**MMR**^**d**^
Burkina Faso	916	14.7	8.6	61.4	51.7	12.6	34.4	95.5	32.3	1012	6.5	1400
Chad	1015	17.5	16.6	61.9	55.9	19.0	58.6	88.6	60.6	1008	4.8	1500
Congo	425	7.1	8.0	59.2	48.7	91.4	73.7	36.3	39.8	606	4.2	940
Côte d’Ivoire	528	7.2	6.9	60.5	58.3	63.5	82.1	63.4	37.8	1500	7.0	1200
Ethiopia	1075	15.6	6.9	60.0	47.9	12.9	57.9	87.2	46.5	724	4.4	871
Ghana	1131	13.0	10.8	62.6	45.3	41.5	69.7	51.8	26.2	2050	3.1	590
Kenya	944	14.9	20.0	62.6	49.2	5.3	62.6	38.1	22.3	992	6.7	1300
Malawi	1105	29.0	20.2	62.1	46.2	4.7	66.1	42.3	59.9	586	14.2	580
Mali	1166	2.1	1.8	64.3	62.0	19.0	41.4	91.6	34.9	878	1.7	630
Namibia	876	13.0	10.7	64.9	39.3	23.3	81.0	49.3	36.4	6410	21.3	370
Senegal	707	2.6	1.1	60.7	56.2	47.1	50.1	75.9	40.5	1535	0.8	1200
South Africa	455	14.2	25.9	60.1	41.5	57.1	81.9	24.9	49.4	10132	15.6	340
Swaziland	719	4.9	6.8	62.5	51.5	18.2	49.1	47.0	46.3	4053	38.8	370
Zambia	700	17.7	10.6	61.2	47.6	22.3	64.9	36.8	16.8	806	15.6	870
Zimbabwe	885	18.1	6.9	63.1	43.6	24.5	64.8	24.3	26.4	-	24.6	610

^a^At least one of: angina, arthritis, diabetes, or asthma.

^b^GNI per capita in 2002 (in 2005 purchasing power parity adjusted internationally dollars).

^c^Estimated HIV prevalence in the population age 14–49.

^d^Number of deaths of women from pregnancy-related causes per 100,000 live births.

**Figure 2:**
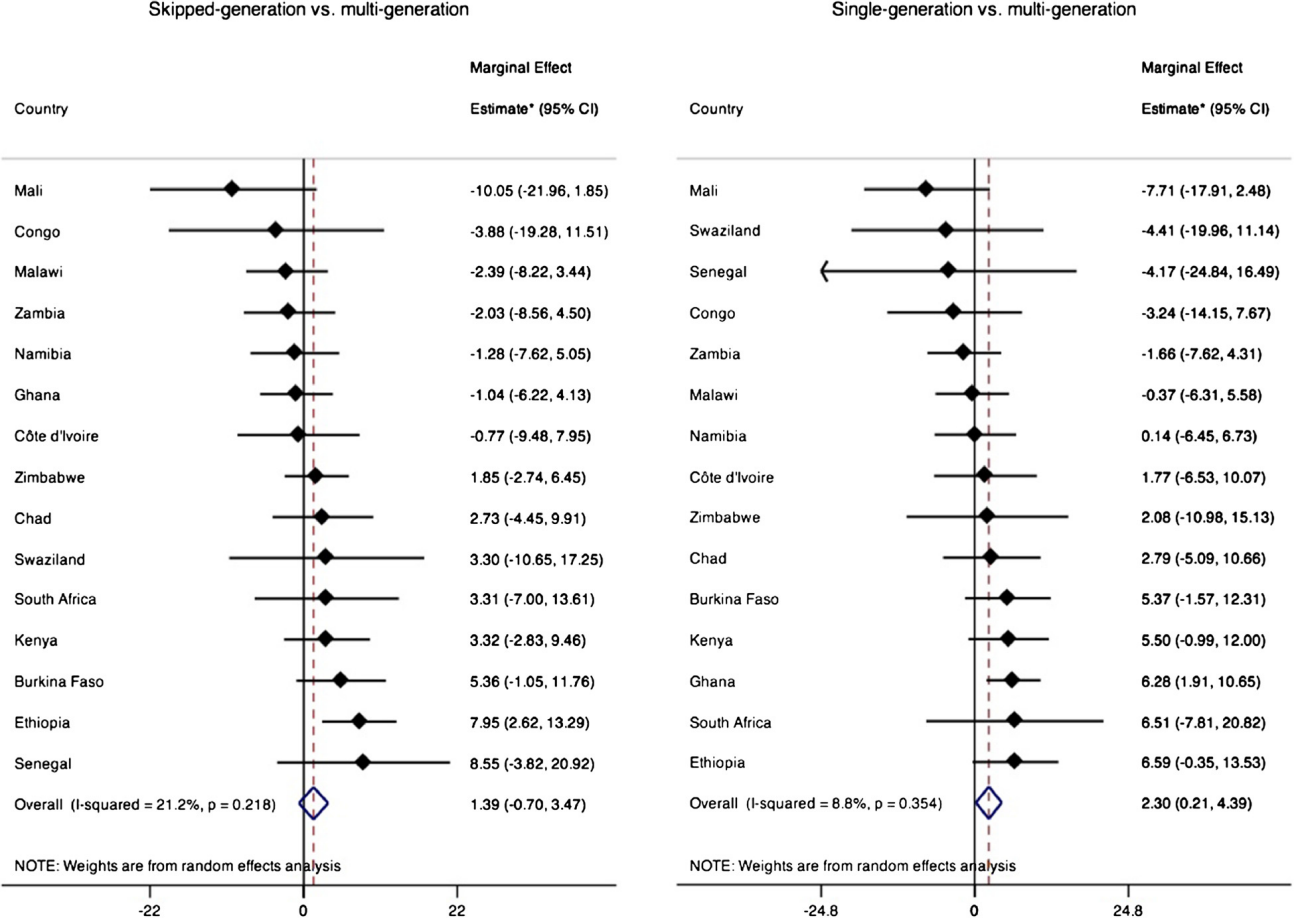
Estimated effects of skipped-generation and single-generation living arrangements on the predicted prevalence of depressive symptoms.

The estimated crude effects of skipped- and single- generation living arrangements compared to multigenerational living arrangement on the predicted prevalence of depressive symptoms were 2.9 (95% CI: 0.6, 5.3) and 3.6 (1.3, 6.0), respectively. The country-specific estimates from multivariate models that adjust for potential confounders are presented in Additional file 1: Table S1 and summarized in Figure 2. The covariate-adjusted random-effects pooled prevalence of depressive symptoms was 2.3 percentage points (95% CI: 0.2, 4.4) higher among individuals living in single-generation households compared to those living in multigenerational households. Living in a skipped generation household compared to a multigenerational household was also associated with higher prevalence of depressive symptoms (1.4 points, 95% CI: -0.7, 3.5), although the difference was smaller than for single-generation households and the confidence interval includes the null. The *I*^2^ statistics for the effects of both skipped-generation and single-generation household living arrangements were low (21.2% and 8.8%, respectively), suggesting that most of the residual variation was due to within-study sampling variability rather than between-country heterogeneity. In addition to the random-effects models, we estimated an overall multivariate model with country fixed effects and found the following variables to be positively associated with depressive symptoms: chronic disease, lower SES, female, and living in a non-multigenerational household (Additional file 1: Table S2).

Table 2 contains estimates from the meta-regression models and provides some evidence that, conditional on individual-level covariates, higher MMR and HIV/AIDS prevalence may be positively associated with the predicted prevalence of depressive symptoms. There was an estimated increase of 0.52 percentage points (95% CI: -0.13, 1.16) in the prevalence of depressive symptoms for each 1/1000-death increase in the rate of maternal mortality and an increase of 0.21 percentage points (95% CI: -0.07, 0.50, respectively) for each 5% rise in HIV/AIDS prevalence, although neither estimate was statistically different from zero. GNIpc did not have a detectable effect on the prevalence of depressive symptoms and overall the country variables explained a very small proportion of between-country variability in depressive symptom prevalence (*R*^2^ = 0.07). Finally, we found little evidence that any of the country-level variables explained a significant proportion of heterogeneity between countries in the effects of living arrangement on the predicted prevalence of depressive symptoms.

**Table 2:** Random-effects meta-regression estimates

**Country variables**	**Meta-regression of country variables on the predicted prevalence of depressive symptoms**	**Meta-regression of country variables on the effect of single-generation living arrangement on the predicted prevalence of depressive symptoms**
	Coefficient (95% CI)	Coefficient (95% CI)
HIV prevalence (%)	0.20 (−0.07, 0.50)	−0.21 (−0.65, 0.22)
log(GNIpc^a^)	−0.47 (−3.79, 2.85)	0.03 (−4.59, 4.66)
Maternal mortality ratio^b^	0.52 (−0.13, 1.16)	0.07 (−0.71, 0.86)
I-squared	80.1%	16.6%

^a^GNI per capita in 2002 (in 2005 purchasing power parity adjusted internationally dollars).

^b^Number of deaths of women from pregnancy-related causes per 100,000 live births.

## Discussion

The primary aim of this study was to assess whether older adults living in single- or skipped-generation households had an increased prevalence of depressive symptoms compared to those living with at least one working-age adult. Controlling for individual-level covariates, we found evidence that older adults living in single-generation households had a 2.3 percentage point higher prevalence of depressive symptoms than those living with at least one working-age adult. This corroborates evidence from many other populations that older people who live alone are more likely to suffer from symptoms of loneliness and depression [1,14,15]. We did not detect a significant increase in depressive symptoms among older Africans living in skipped-generation households compared to those in multi-generational households. Well-established risk factors for depression among older adults, including being female, having a chronic disease, and having low SES, were also identified as risk factors for depressive symptoms in our study [16].

Our results are consistent with previous studies in low-income settings that have reported that living with children compared to living alone offers some protection from depressive symptoms, even in the absence of working-age adults [14,15]. One study from rural China found that older adults living in skipped- compared to three-generation households had higher scores on a depression scale; however, older adults in single- compared to skipped-generation households were almost twice as likely to have depressive symptomatology [15]. Findings of the Chinese study were primarily explained by the higher remittances received by older people in skipped generation households from their absent adult children, who had most often migrated to urban areas for work. Unfortunately, in our study we were unable to determine the orphan status of children in skipped-generation households or whether the households were receiving material support from absent adult children.

We also attempted to explain whether differences across countries in both the prevalence of depressive symptoms and the magnitude of the associations between living arrangements and depressive symptoms were related to country-level indicators of health and economic status. Although there was substantial heterogeneity across countries in the prevalence of depressive symptoms, the country-level variables did not explain much of this heterogeneity. There was some evidence that older adults in countries with higher rates of HIV/AIDS and maternal mortality might experience a higher prevalence of depressive symptoms, but the effects were generally weak and imprecisely estimated. With more countries or larger sample sizes we may well have had the precision to detect important effects for some of the country variables. Differences across countries in the estimated associations between living arrangements and depressive symptoms were more the result of sampling variability than any inherent heterogeneity potentially explainable by country-level variables. It is therefore unsurprising that none of the country variables explained the heterogeneity. Nevertheless, it is possible that our study missed certain country-level factors that may also have been important to consider. For example, countries with universal old age pension programs may provide better economic security that could help mitigate the detrimental effects of solitary or skipped-generation living arrangements on the depressive symptoms of older adults. In this study, we were unable to examine this variable since only South Africa and Namibia had pension programs at the time of the WHS. However, as several other countries have developed programs over the past decade, we think this is an interesting and policy-relevant question for future research.

Several limitations need to be considered in interpreting our results. First, the WHS was a large survey designed to collect information on individual health and health system interactions. Assessing mental health outcomes was not a main focus of the WHS. Thus, our measure of depressive symptoms was restricted to the seven questions available in the WHS, which, although taken from a previously validated depression scale, has not itself been validated. Nevertheless, our measure includes hallmark depressive symptoms, considers important duration and frequency criteria, and was used previously in at least one highly-cited study [18]. There are also some limitations of our exposure measure. According to our living arrangement definitions, a household where an older person is caring for a sick adult child would have been classified as multi-generational, although the older person may be less likely to receive the material and instrumental support that would presumably make a multi-generational living arrangement more protective against depressive symptoms. We also did not have information on whether the older person had experienced the death of an adult child or another traumatic event that may have affected their responses at the time of the survey. Data that can directly address the older person’s support systems and traumatic life events will be important in further research investigating the impact of living arrangements on the health of older persons. Finally, our analysis is based on cross-sectional data and, as a consequence there is ambiguity regarding the temporal relationship between living arrangements and depressive symptoms. This issue of reverse causality has been shown to lead to underestimation of the protective effects of coresidence with an adult child on depressive symptoms among older widowed women in South Korea [35].

## Conclusions

Although the proportion of older people sub-Saharan Africa is still relatively small compared to other world regions, this is expected to double from 5% to 10% between 2010 and 2050, a relative increase greater than any other global region [1]. This intensifies concerns surrounding the health and welfare of the growing number of older people living alone and in skipped-generation households. In this study, we provide evidence that older people living in single-generation households report a higher prevalence of depressive symptoms compared to those living with at least one working-age adult. As depressive symptoms are known to be predictive of poor quality of life and increased morbidity and mortality, it is important to identify population groups that may be especially vulnerable. Work remains to be done to further our understanding of the complex relationship between living arrangements, support systems, and depressive symptoms and understand what policies can be put in place to mitigate the potential detrimental effects of solitary living on the mental health of older persons in sub-Saharan Africa.

## Competing interests

The authors declare that they have no competing interests.

## Authors’ contributions

SH and BM conceptualized the paper and developed the analysis plan. BM performed the analysis and wrote the first draft. All authors contributed to the interpretation of findings, critically reviewed the manuscript for important intellectual content and approved the final version.

## Pre-publication history

The pre-publication history for this paper can be accessed here:

http://www.biomedcentral.com/1471-2458/13/682/prepub

## Supplementary Material

Additional file 1: Table S1 Marginal effects and standard errors of covariates on the predicted prevalence of depressive symptoms estimated from country-specific multivariate logistic regression models. **Table S2.** Average marginal effects and standard errors of covariates on the predicted prevalence of depressive symptoms estimated from an overall multivariate logistic regression model with country fixed effects. Click here for file
