# Supplementary material for: The relationship of living arrangements and depressive symptoms among older adults in sub-Saharan Africa
Source: BMC Public Health. 2013 Jul 25;13:682. doi: 10.1186/1471-2458-13-682 (PMC3737026; doi:10.1186/1471-2458-13-682)
Supplement: Additional file 1: Table S1 — Marginal effects and standard errors of covariates on the predicted prevalence of depressive symptoms estimated from country-specific multivariate logistic regression models. Table S2. Average marginal effects and standard errors of covariates on the predicted prevalence of depressive symptoms estimated from an overall multivariate logistic regression model with country fixed effects. [file 1471-2458-13-682-S1.doc]

Additional file

Table S1: Marginal effects and standard errors of covariates on the predicted prevalence of depressive symptoms estimated from country-specific multivariate logistic regression models

| Country | Skipped generation household ^a^ | Single generation household ^a^ | Age (5 year increase) | Male | No education | Global wealth index | Urban | Married ^b^ | Chronic disease ^c^ |
| --- | --- | --- | --- | --- | --- | --- | --- | --- | --- |
| Burkina Faso | 5.36  (3.27) | 5.37  (3.54) | 1.80  (0.64) | -1.21 (2.96) | -6.03 (10.55) | -0.38  (1.92) | -1.84 (4.34) | -3.84  (3.61) | 12.73 (2.80) |
| Chad | 2.72  (3.66) | 2.79  (4.02) | 2.52  (0.76) | -9.36 (4.19) | 1.66  (6.75) | -4.44  (6.30) | -1.66 (4.27) | 0.14  (3.66) | 14.37 (3.42) |
| Congo | -3.88  (7.86) | -3.24  (5.57) | 0.02  (1.59) | 2.63 (5.27) | 7.85  (5.93) | 2.30  (6.19) | 4.62 (5.71) | -8.57  (4.81) | 19.71 (4.82) |
| Côte d’Ivoire | -0.77  (4.45) | 1.77  (4.24) | -0.40  (0.93) | -3.07 (3.74) | 4.07  (3.87) | -1.07  (3.06) | 11.15 (4.22) | 3.62  (3.87) | 9.32 (3.93) |
| Ethiopia | 7.95  (2.72) | 6.61  (3.54) | 1.37  (0.61) | 0.31 (3.16) | 8.21  (7.14) | -7.65  (3.09) | 5.73 (5.31) | -0.16  (3.31) | 10.29 (2.43) |
| Ghana | -1.04  (2.64) | 6.28  (2.23) | 0.12  (0.54) | -1.95 (2.45) | -0.31 (2.26) | -7.51  (2.02) | 5.99 (2.40) | -0.92  (2.49) | 4.40 (2.30) |
| Kenya | 3.32  (3.14) | 5.51  (3.31) | -1.06  (0.71) | -8.38 (3.80) | 1.87  (3.10) | -1.93  (3.85) | -2.34 (6.16) | -2.62  (2.97) | 8.21 (3.03) |
| Malawi | -2.39  (2.97) | -0.37  (3.03) | 0.02  (0.69) | -5.50 (3.04) | 4.04  (2.76) | -8.53  (5.12) | -5.60 (5.69) | -2.39  (3.06) | 6.05 (3.08) |
| Mali | -10.05  (6.07) | -7.71  (5.20) | -0.08  (0.44) | -3.50 (2.21) | -4.51 (3.35) | 0.76  (1.68) | 2.22 (2.52) | -1.97  (2.37) | 11.35 (2.29) |
| Namibia | -1.28  (3.23) | 0.13  (3.36) | 0.39  (0.51) | -1.58 (2.99) | 9.94  (3.60) | 1.26  (1.99) | -0.28 (3.60) | -2.35  (2.80) | 6.19 (2.55) |
| Senegal | 8.56  (6.31) | -4.17  (10.54) | 0.79  (0.81) | -1.97 (4.03) | -2.46 (4.70) | -2.68  (2.98) | -3.36 (4.54) | -1.13  (4.30) | 6.47 (3.78) |
| South Africa | 3.31  (5.26) | 6.51  (7.31) | -0.17  (1.30) | -11.95 (6.54) | -5.70 (5.37) | -17.57  (11.92) | -0.16 (4.19) | -4.27  (5.60) | 0.92 (4.22) |
| Swaziland | 3.30  (7.11) | -4.44  (7.93) | -0.23  (1.25) | -5.00 (4.94) | -0.13 (5.40) | -62.40  (56.81) | 1.12 (6.38) | 2.53  (6.44) | 6.77 (5.09) |
| Zambia | -2.03  (3.33) | -1.66  (3.04) | -0.04  (0.80) | 1.56 (3.39) | 5.99  (3.29) | 2.96  (1.80) | -7.31 (3.98) | -2.13  (2.69) | 7.55 (2.80) |
| Zimbabwe | 1.85  (2.34) | 2.08  (6.66) | 1.07  (0.91) | 0.34 (2.91) | -3.73 (4.27) | 0.33  (2.54) | -5.92 (4.51) | -3.06  (3.46) | 1.27 (2.94) |

^a^ Reference group: multigenerational household

^b^ Reference group: single/divorced/widowed

^c^ At least one of: angina, arthritis, diabetes, or asthma

Table S2: Average marginal effects and standard errors of covariates on the predicted prevalence of depressive symptoms estimated from an overall multivariate logistic regression model with country fixed effects

|  | Average marginal effect  (95% confidence interval) |
| --- | --- |
| Living arrangement |  |
| Multigenerational | (ref) |
| Skipped-generation | 4.2 (1.5, 6.9) |
| Single generation | 5.2 (-1.6, 12.0) |
| Age (5 year increase) | 0.4 (-0.2, 1.1) |
| Male | -3.8 (-6.6, -0.9) |
| No education | 0.1 (-3.2, 3.4) |
| Global wealth index | -4.2 (-7.1, -1.4) |
| Married ^a^ | -2.2 (-.5.2, 0.9) |
| Urban residence | 1.5 (-2.8, 5.7) |
| Chronic disease ^b^ | 6.8 (3.8, 9.8) |
| Country |  |
| Burkina Faso | (ref) |
| Chad | 7.7 (3.3, 12.1) |
| Congo | 2.3 (-3.9, 8.6) |
| Côte d'Ivoire | -4.8 (-9.3, -0.3) |
| Ethiopia | 0.2 (-3.5, 3.9) |
| Ghana | -2.7 (-6.3, 1.0) |
| Kenya | -1.3 (-5.5, 2.9) |
| Malawi | -3.1 (-7.0, 0.9) |
| Mali | -5.2 (-8.7, -1.7) |
| Namibia | -4.7 (-8.5, -0.9) |
| Senegal | -3.4 (-7.6, 0.8) |
| South Africa | -3.5 (-9.6, 2.5) |
| Swaziland | 3.8 (-2.1, 9.6) |
| Zambia | -.4.9 (-8.8, -0.9) |
| Zimbabwe | -7.3 (-11.9, -2.6) |

^a^ Reference group: single/divorced/widowed

^b^ At least one of: angina, arthritis, diabetes, or asthma
